# Early postnatal defects in neurogenesis in the 3xTg mouse model of Alzheimer’s disease

**DOI:** 10.1038/s41419-023-05650-1

**Published:** 2023-02-18

**Authors:** Yubing Liu, Maria Bilen, Marie-Michelle McNicoll, Richard A. Harris, Bensun C. Fong, Mohamed Ariff Iqbal, Smitha Paul, Janice Mayne, Krystal Walker, Jing Wang, Daniel Figeys, Ruth S. Slack

**Affiliations:** 1 Department of Cellular and Molecular Medicine, University of Ottawa Brain and Mind Research institute, K1H 8M5 Ottawa, Canada; 2 Ottawa Institute of Systems Biology and Department of Biochemistry, Microbiology and Immunology, Faculty of Medicine, University of Ottawa, K1H 8M5 Ottawa, Canada; 3 Regenerative Medicine Program, Ottawa Hospital Research Institute, K1H 8L6 Ottawa, Canada

**Keywords:** Adult neurogenesis, Neural stem cells

## Abstract

Alzheimer’s disease (AD) is a progressive neurodegenerative disorder leading to dementia. The hippocampus, which is one of the sites where neural stem cells reside and new neurons are born, exhibits the most significant neuronal loss in AD. A decline in adult neurogenesis has been described in several animal models of AD. However, the age at which this defect first appears remains unknown. To determine at which stage, from birth to adulthood, the neurogenic deficits are found in AD, we used the triple transgenic mouse model of AD (3xTg). We show that defects in neurogenesis are present as early as postnatal stages, well before the onset of any neuropathology or behavioral deficits. We also show that 3xTg mice have significantly fewer neural stem/progenitor cells, with reduced proliferation and decreased numbers of newborn neurons at postnatal stages, consistent with reduced volumes of hippocampal structures. To determine whether there are early changes in the molecular signatures of neural stem/progenitor cells, we perform bulk RNA-seq on cells sorted directly from the hippocampus. We show significant changes in the gene expression profiles at one month of age, including genes of the Notch and Wnt pathways. These findings reveal impairments in neurogenesis very early in the 3xTg AD model, which provides new opportunities for early diagnosis and therapeutic interventions to prevent neurodegeneration in AD.

## Introduction

Alzheimer’s disease (AD) is the most common cause of dementia [1]. At present, more than 100 years after its discovery, there is still no effective treatment for individuals with AD [2]. The symptoms of the disease include progressive memory loss and learning difficulties, consistent with synaptic impairment eventually leading to extensive neuronal death [1]. AD is characterized by heavy cortical and hippocampal shrinkage, and severe ventricle enlargement on a macroscopic scale [1, 2]. At the cellular level, multiple gene expression changes have been linked to early onset of the disease: (a) the amyloid precursor protein (APP), a gene encoding a transmembrane protein that gets cleaved forming amyloid-beta (Aβ) which then accumulates and forms extracellular plaques; (b) Presenilin1 (PSEN1), subunits of the γ-secretase enzyme responsible for the cleavage of APP; (c) The microtubule-associated protein tau (MAPT), a gene encoding for the scaffold protein tau which tends to get hyperphosphorylated in AD and form neurofibrillary tangles [3].

The hippocampus, one of the regions where the neuronal loss in AD is observed, is an important region for learning and memory [4]. The subgranular zone (SGZ) of the dentate gyrus of the hippocampus represents a well-characterized neurogenic niche in the mammalian brain, along with the subventricular zone (SVZ) of the lateral ventricles. Neurogenesis involves the generation of new functional neurons from a pool of neural stem cells (NSC) [5]. In the SGZ of adult mice neurogenesis facilitates the formation of granule neurons which contribute to hippocampal memories and play a key role in learning [6]. A decrease in neurogenesis is correlated with aging and cognitive decline. Similarly, patients at early stages of AD have lower levels of new-born neurons suggesting that the defect in adult hippocampal neurogenesis may potentially be used as an early marker of disease progression [5, 7].

To study the etiology and progression of AD, several mouse models carrying human mutations observed in genes associated with familial, early-onset form of the disease have been created [3]. These models attempt to recapitulate the formation of extracellular Aβ plaques and/or the intracellular tau neurofibrillary tangles [3]. Of these models, both J20 and TgCRND8 mice carry the Swedish and Indiana mutations of the human APP which exhibit Aβ accumulation, plaque formation and synaptic dysfunction, but do not recapitulate the formation of neurofibrillary tangles [3, 8]. The 3xTg mouse model carries 3 human mutant genes for APP, MAPT and PS1, associated with familial AD and shows Aβ accumulation and tau pathologies starting at 6 months of age [9].

While a decline in hippocampal neurogenesis has been described in AD, it remains unknown when these deficits begin and whether a causative mechanistic link exists with AD neuropathology [5, 10]. Adverse effects of A*β* pathology on neural stem cells, and the subsequent decline in adult neurogenesis, could potentially contribute to the cognitive impairment and the depletion of neurons observed in AD [7]. Indeed, in several animal models of AD a defect in NSC population is observed earlier than the appearance of Aβ plaques [11, 12]. For example, the formation of Aβ plaques and tau pathology starts at 6 months in the 3xTg model, while cognitive function deteriorates by 4 months along with defects in SGZ neurogenesis [9, 13]. Nonetheless, it remains unclear whether changes in adult neurogenesis are an early event in AD neurodegeneration or a later consequence of the disease [10]. Identifying early changes in adult neurogenesis may be of clinical relevance for diagnostic purposes [5, 14], and may permit intervention in presymptomatic at-risk individuals within a therapeutic window that could allow for a better prognosis [15, 16].

Many studies have focused on assessing neurogenesis in the 3xTg mice at the adult stage. However, earlier changes may also play an important role in cognitive decline. Here, we show that defects associated with adult neurogenesis begin at early postnatal stages and precede the onset of AD neuropathology. RNA-seq analyses on FACS-isolated adult neural stem and progenitor cells directly from the hippocampus further support these findings by revealing striking molecular changes as early as 1 month of age. Importantly, our results suggest that such changes are detectable well before the onset of disease pathology which opens potential opportunities for early intervention therapies.

## Materials and methods

### Mice

The triple-transgenic mice (3xTg) were obtained from the Jackson Laboratory and generated at the University of California Irvine, USA [9] (JAX stock number: 004807). Wild-type mice from the same genetic background were maintained as an independent colony and served as a non-transgenic control (NTG, JAX stock number: 101045). The Nestin-CreER^T2^;R26LSL-EYFP^STOP^ mouse line (Nestin-Cre) was a gift from Dr. Suzanne J Baker at the University of Tennessee Health Science Center [17, 18]. Nestin-CreER^T2^;R26LSL-EYFP^STOP^ mice bear the CreER^T2^ transgene that is driven by a Nestin promoter and an enhancer in the second intron, an internal ribosomal entry site and a human placental alkaline phosphatase, and harbor the R26LSL-EYFP^STOP^ transgene that maps Cre-activity in YFP-positive population [19, 20]. 3xTg or NTG mice were cross-bred with Nestin-CreER^T2^;R26LSL-EYFP^STOP^ mice to generate mice with conditional YFP labeling of Nestin-positive cells, named 3xTg;Nestin-YFP or NTG;Nestin-YFP. Animal experiments were approved by the University of Ottawa’s Animal Care Committee, which abides by the guidelines of the Canadian Council on Animal Care. Genotyping was conducted according to the strain recommendation.

In all experiments both males and females were used unless indicated otherwise. For embryonic studies, the day of the plug detection was considered as embryonic day 0.5 (E0.5). For postnatal studies, the day the pups were born was considered as postnatal day 0 (P0).

### EdU incorporation

In vivo labelling of proliferating cells was achieved by giving an intraperitoneal injection of 50 μg of EdU (5-ethynyl-2’-deoxyuridine) per gram of body weight (Clickbase, BCK647-IV-IM- M) 2 h prior to being sacrificed. For the embryonic brains, pregnant mothers were given a single injection of EdU 24 hours prior to the harvest. EdU staining was conducted in accordance with the manufacturer’s instructions.

### Tissue processing

Adult brain tissues were processed as described before [21]. Briefly, mice were perfused with of 4% cold paraformaldehyde (PFA, pH 7.4) and brains were post-fixed in 4% PFA at 4 °C for 24 h. For E15.5, P0, P5, and P7 time points, mice were dissected, decapitated, and stored in 4% PFA at 4 °C for 48 h. Brains were then incubated and stored in 20% sucrose in PBS at 4 °C. Adult and postnatal brains were frozen and sectioned as free-floating serial coronal sections of 30*μm* thickness in a 1 in 9 and 1 in 6 series, respectively, to reveal all hippocampal structures. E15.5 brains were sectioned at 20*μm* thickness onto Superfrost Plus slides (Fisher) in a 1 in 6 series.

### Immunostaining and histology

Immunofluorescence staining and cresyl violet were performed as described before [21, 22]. Briefly, every ninth or sixth section throughout the dentate gyrus was used to perform the staining. For the immunofluorescence, the sections were incubated with a combination of primary antibodies in PBS supplemented with 0.1% TritonX and Tween20. The primary antibodies used were Sox2 (GT15098, Neuromics), DCX (4604 S, Cell Signaling Technology), Hopx (HPA030180, Atlas), S100b (PA5-78181, Invitrogen) and the phosphorylated histone H3 (Ser10) (pHH3, 06-570, Millipore). For the Cresyl violet stain, slides were dehydrated and rehydrated using alcohol gradient and then stained with 0.25% of Cresyl violet in 200 mM of acetate buffer. Images were acquired with DeltaVision Elite-Olympus IX-71 or Zeiss AxioScan Z1 at a 20X magnification. Images were then processed and quantified with Fiji [23].

### Cell counts and volume measurements

Cells expressing the markers of interest described above were quantified from every ninth or sixth section throughout the SGZ. The total counts from one hemisphere were multiplied by 9 or 6 to generate an estimate of the total cell number in the dentate gyrus. For Cresyl Violet staining, the area of the granular cell layer (GCL), dentate gyrus (DG), hippocampus, and whole hemisphere were traced and multiplied by the thickness of the slice for each section (30 *μm*) and by 9 or 6 to get the volume of the entire brain structure [21, 24]. The cortical thickness is measured at the level of the parietal association cortex and secondary visual cortex. Images were quantified with Fiji (Image J) [23].

### Statistical analysis

An unpaired, two-tailed Student’s *t*-test was performed between genotypes (3xTg and NTG). Analysis was performed using GraphPad PRISM software (GraphPad Software, Inc). Biological replicates ‘n’ for each experiment were shown in figure legends.

### Tamoxifen administration

Cre recombination was induced by administration of tamoxifen (TAM) through oral gavage at a dose of 120 mg/kg daily for five consecutive days on 3xTg;Nestin-YFP and NTG;Nestin-YFP mice at 3-4 weeks old [21].

### Fluorescence-activated cell sorting (FACS) for Bulk RNA-seq

Neural stem cells (NSCs) were isolated from the juvenile SGZ as described before [25, 26]. Briefly, the SGZ were dissected coronally in ice-cold artificial cerebrospinal fluid (ACSF) from 3xTg;Nestin-YFP or NTG;Nestin-YFP mice 10 days after the last dose of tamoxifen. Tissue was homogenized and digested in pre-warmed papain solution at 20 U/ml (Worthington Cat. PAP3126) for 10 min at 37 °C on a rotator. An equal volume of resuspension medium that contains 0.5 mg/ml of DNase I (Roche Cat. # 11284932001) and 10% of fetal bovine serum (Wisent Cat. # 080150) was added to the digested tissue. The samples were triturated 4 to 6 times with P1000 micropipet and incubated for 5 min at room temperature. 3.9 ml of cell suspension was gently mixed with 1.1 ml of 90% Percoll in PBS. Cells were collected at 500 x *g* for 12.5 min at 4 °C. Cells were resuspended in sorting medium (DMEM:F12, 1 mM EDTA, 3 μM Propidium Iodide). Sorted YFP-positive and PI-negative cells were collected in DMEM:F12 plus 2% B-27 (ThermoFisher, Cat.# 12587010).

### RNA extraction, RT-PCR and primers

RNA was extracted using RNeasy Micro Kit (Invitrogen, Cat. 74004) following the manufacture’s manual. RNA from 3 biological replicates was sent to Toronto SickKids, The Center for Applied Genomics. cDNA library was established using Takara SMART-Seq V4 cDNA synthesis / Nextera XT library prep and sequenced on NovaSeq SP flowcell SR200.

### Bulk RNA-seq analysis

Fastq files were aligned to GRCm38 index using hisat2 in R studio [27, 28]. Reads were assigned using featureCounts, part of the subread package [29]. DESeq2 was used to establish differential gene expression between NTG and 3xTg [30]. Genes were considered significantly different between two genotypes if they meet the following criteria: 1) adjusted *p*-value less than 0.05, and 2) log2 fold change > 0.5 or < -0.5. Gene ontology analysis was performed using DAVID Bioinformatics [31].

### Quantitative PCR analysis

Quantitative PCR was calculated using ΔΔCt method [32]. The geometric mean of Sdha and Appl2 expression served as the normalization control. Each of the replicates was normalized to the average of NTG to have comparable presentation of the results. An unpaired and two-tailed Student’s *t*-test was performed on 7 independent biological replicates. The chart was generated using Graphpad Prism 8 (GraphPad Software). Primers were listed in Table S1.

## Results

### Reduced volume of hippocampal structures in 3xTg mice at early ages

**Fig. 1:**
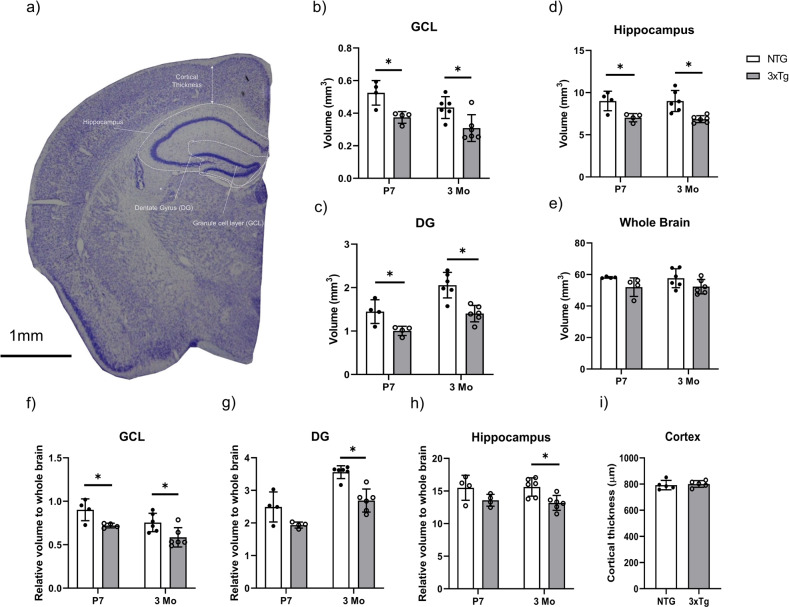
Reduced volume of hippocampal structures in 3xTg mice at early ages. **a** Representative Cresyl violet staining of a coronal section of NTG mice brain. GCL, DG, hippocampus and cortical thickness are labelled. Scale Bar 1 mm. **b** Absolute volume measurements of GCL, **c** DG, **d** hippocampus, and **e** whole brain for NTG and 3xTg at postnatal day 7 (P7) and 3 months of age (3Mo). **f** Relative volume measurements of GCL, **g** DG, **h** hippocampus, to the whole brain for NTG and 3xTg at postnatal day 7 (P7) and 3 months of age (3Mo). **i** Average cortical thickness for NTG and 3xTg at 1 month of age. Data represented as mean ± SD. A two-tailed, unpaired Student’s *t*-test was used for statistical analysis (*p < 0.05), *n* = 3–6 for each group.

To determine if there are early changes in the hippocampus, hippocampal and brain volumes were measured in the 3xTg mouse model and compared to non-transgenic controls of the same genetic background. Volumes were measured at postnatal day 7 (P7) and 3 months of age (3Mo), before extracellular hippocampal Aβ deposit and tangle formation at 6 months [9, 22] (Fig. 1a). The volumes of the GCL, DG, and hippocampus were significantly reduced in 3xTg mice at 3 months, and strikingly, as early as P7 in terms of absolute volume (Fig. 1b, c, d). No difference in whole brain volumes were detected between NTG and 3xTg mice. As a non-neurogenic control region, we also measured cortical thickness and found no significant differences (Fig. 1a, i). Considering these results and the recently published data in human subjects showing reduced numbers of immature neurons in the brains of human AD patients [5], we next asked whether adult neural stem cells and their progeny residing in the SGZ of the hippocampus were also affected at these early time points.

### Early postnatal defects in neurogenesis in the SGZ of 3xTg mice

**Fig. 2:**
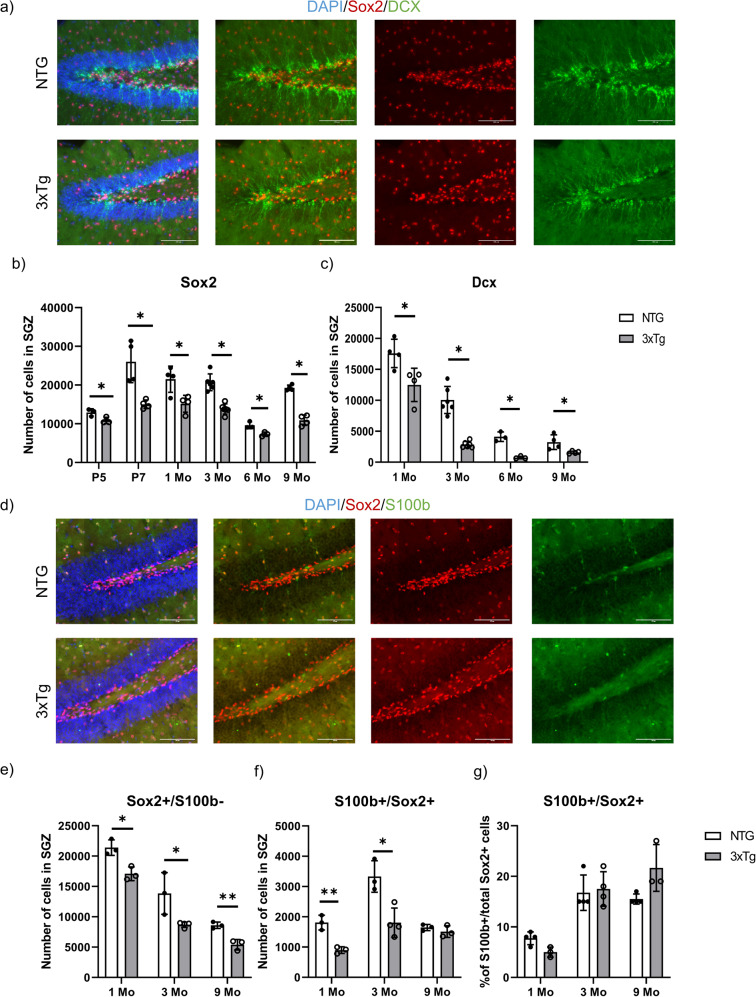
Decline of neural stem/progenitor cells and immature neurons in the SGZ of 3xTg mice at various time points. **a** Representative immunostaining of Sox2 (in red) and DCX (in green) at 1 month of age in coronal sections of NTG (Top) and 3xTg (Bottom). GCL is visualized by DAPI. Scale bar 100 *µm*. **b** Quantification of the total number of Sox2-positive cells in the SGZ at postnatal day 5 (P5); postnatal day 7 (P7); 1 month of age (1Mo); 3 months of age (3Mo); 6 months of age (6Mo); and 9 months of age (9Mo). **c** Quantification of the total number of DCX-positive cells in the SGZ at 1 month of age (1Mo); 3 months of age (3Mo); 6 months of age (6Mo); and 9 months of age (9Mo). **d** Representative immunostaining of Sox2 (in red) and S100b (in green) at 1 month of age in coronal sections of NTG (Top) and 3xTg (Bottom). GCL is visualized by DAPI. Scale bar 100 *µm*. **e** Quantification of the total number of Sox2-positive/S100b-negative cells and **f** Sox2-positive/S100b-positive cells in the SGZ at 1 month of age (1Mo); 3 months of age (3Mo); and 9 months of age (9Mo). **g** Graphs representing the percentage of S100b-positive cells out of the total Sox2-positive cells in the SGZ at 1 month of age (1Mo); 3 months of age (3Mo); and 9 months of age (9Mo). Data represented as mean ± SD. A two-tailed, unpaired Student’s *t*-test was used for statistical analysis (*p* < 0.05*, *p* < 0.01**), **b and c**: *n* = 3 for P5 and 6Mo; *n* = 4 for P7, 1Mo and 9Mo; *n* = 6 for 3Mo. **e, f and g:** *n* = 3 for 1Mo and 9Mo; *n* = 4 for 3Mo.

**Fig. 3:**
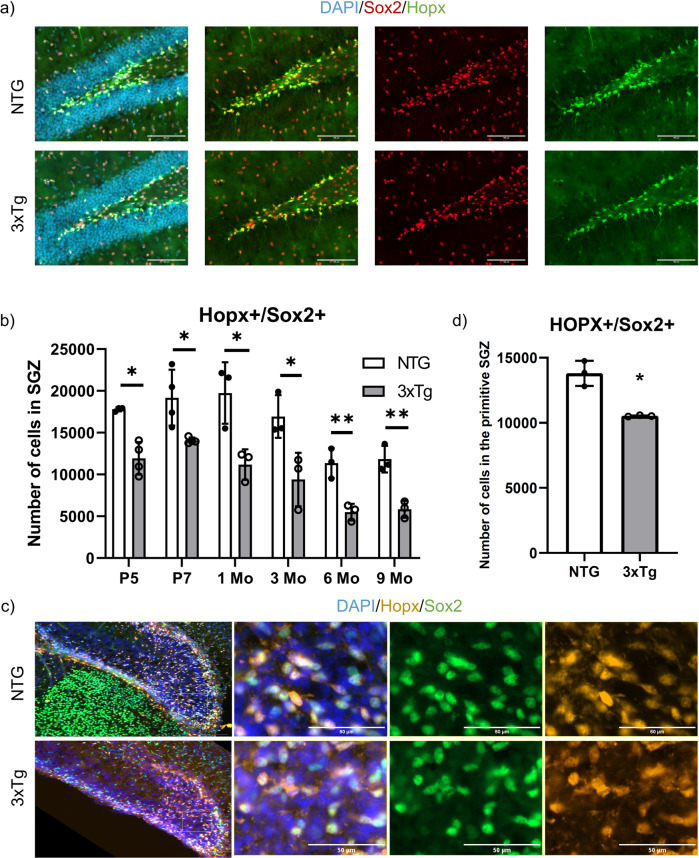
Decline in the Hopx-expressing population in the SGZ of 3xTg mice at as early as postnatal days. **a** Representative fluorescent images of Hopx (in green) and Sox2 (in red) at 1 month of age in coronal section of NTG (Top) and 3xTg (Bottom). GCL is visualized by DAPI. Scale bar 100 *µm*. **b** Quantification of the total number of Hopx-positive/Sox2-positive cells in the SGZ at postnatal day 5 (P5); postnatal day 7 (P7); 1 month of age (1Mo); 3 months of age (3Mo); 6 months of age (6Mo) and 9 months of age (9Mo). **c** Representative fluorescent images of Hopx (in yellow), Sox2 (in green) and DAPI (Blue) at postnatal day 0 in coronal section of NTG (Top) and 3xTg (Bottom). Scale bar 100 µm. Magnified view of fluorescent images were shown for merged, Sox2 and Hopx on the left. **d** Quantification of the total number of Hopx-positive/Sox2-positive cells in the primitive structure of the dentate gyrus at postnatal day 0. Data represented as mean ± SD. A two-tailed, unpaired Student’s *t*-test was used for statistical analysis (*p* < 0.05*, *p* < 0.01**), *n* = 4 for P5 and P7; *n* = 3 for P0, 1Mo, 3Mo, 6Mo and 9Mo.

To measure the number of neural stem/progenitor cells in the SGZ, we used well-established markers of the Type 1 (radial glial stem-like cell) and Type 2 progenitor cells. As Sox2 is expressed in both populations, we first quantified the total Sox2-expressing cells in the SGZ of both 3xTg and NTG mice at P5, P7, 1, 3, 6 and 9 months of age (Figs. 2a, 2b). Strikingly, Sox2-positive NSCs were significantly reduced in 3xTg mice as early as P5 (15.9 ± 5.6% reduction), and P7 (42.2 ± 10.6% reduction), as well as at later ages, 1, 3, 6, and 9 months (Figs. 2a, b). To exclude Sox2-expressing astrocytes, we performed double labelling with Sox2 and S100b, a marker of mature astrocytes. A significant decrease in the Sox2-positive/S100b-negative cells was detected starting at 1 month (Fig. 2e) while no significant changes were found in the percentage of S100b-positive astrocytes co-labeled with Sox2 (Figs. 2f, g). To further confirm these findings, we double-labeled cells with Sox2 and Hopx, which labels the quiescent Radial Glia-like cells (Type 1, RGL) previously identified to specify the adult SGZ neurogenic NSCs [33]. Interestingly, Hopx/Sox2-double-labelled NSCs were significantly reduced in 3xTg mice as early as P5 ((32.9 ± 6.5 % reduction), and P7 (26.6 ± 8.8% reduction), as well as at 1, 3, 6, and 9 months (Figs. 3a, b). Together these results support the early deficit of NSCs in the SGZ neurogenic niche of 3xTg animals.

To determine whether there is a reduction in the number of new-born neurons, we quantified Doublecortin (Dcx)-positive cells, a marker of newly committed neuroblasts [34]. Dcx-expressing cells were significantly decreased as early as 1 month of age (29 ± 10% reduction) and at 3 months of age (71.8 ± 9.1% reduction) in the 3xTg SGZ (Figs. 2a, 2c). Together our results reveal early deficits in the hippocampal neurogenic niche of the 3xTg mice, with a decrease in NSCs as early as P5, and a reduction in neurogenesis detected at 1 month of age.

Given the early postnatal defect found in the SGZ of 3xTg mice, we next asked whether a similar deficit in neurogenesis occurred during late embryonic development. Hopx has been previously identified as a marker of neural stem and progenitor cells, which appears in the dentate neuroepithelium (DNe) at E11.5 [33]. The proliferating precursors expressing Hopx in the primitive dentate give rise to granule neurons, and transit into RGL neural progenitors during early postnatal life [33]. We examined Hopx-positive cells at P0 and E15.5. Hopx/Sox2-positive population was significantly decreased in the developing dentate at P0 (23.7 ± 4% reduction) (Figs. 3c, 3d). However, at E15.5, neither the total nor the proliferating Hopx-positive cells was affected in the DNe and the dentate migratory stream (DMS) (Figure S1) [35]. These results suggest that the deficits in adult neurogenesis observed in 3xTg mice likely occur postnatally, as the hippocampal neurogenesis peaks neonatally [36].

### Reduced proliferation of NSCs at early postnatal age

**Fig. 4:**
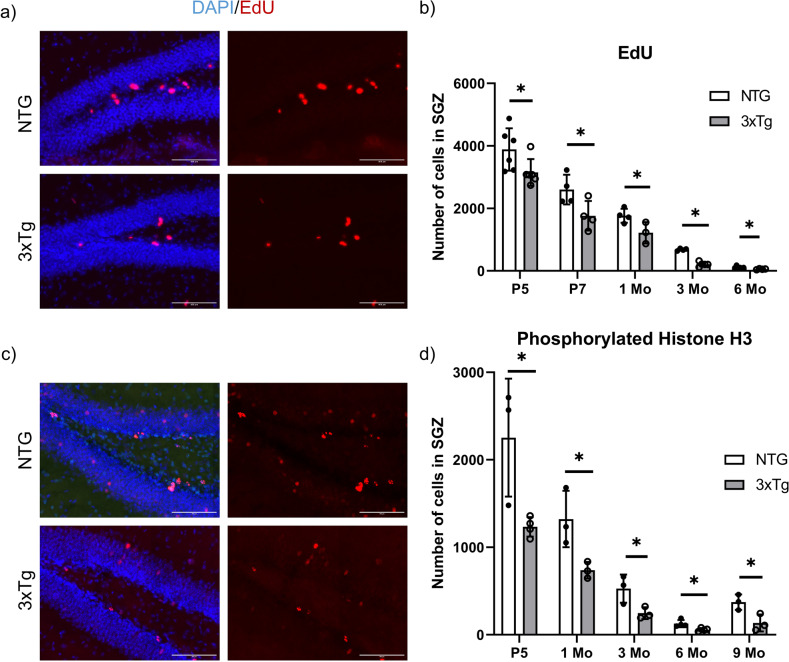
Reduced proliferation of neural stem/progenitor cells in the SGZ of 3xTg mice at as early as postnatal days. **a** Representative fluorescent images of EdU staining in the SGZ after 2 hours of incorporation. Scale bar 100 *µm*. **b** Quantification of EdU-positive cells in the SGZ of NTG and 3xTg mice at multiple time points. postnatal day 5 (P5); postnatal day 7 (P7); 1 month of age (1Mo); 3 months of age (3Mo) and 6 months of age (6Mo). **c** Representative fluorescent images of pHH3 staining in the SGZ of NTG and 3xTg mice at multiple time points. Scale bar: 100 µm. **d** Quantification of pHH3-positive cells in the SGZ of NTG and 3xTg mice at multiple time points. postnatal day 5 (P5); 1 month of age (1Mo); 3 months of age (3Mo); 6 months of age (6Mo) and 9 months of age (9Mo). Data represented as mean ± SD. Statistical analysis was performed with an unpaired two-tailed Student’s *t*-test (*p* < 0.05*, *p* < 0.01**). **b** *n* = 6 for P5; *n* = 4 for P7; *n* = 3 - 4 for 1Mo and 6Mo; and *n* = 4 - 5 for 3Mo. **d** *n* = 3.

Considering the early reduction in the volume of hippocampal structures, as well as in the numbers of Sox2/Hopx-expressing NSCs and newly committed Dcx-expressing neuroblasts, we determined whether there was a decrease in proliferation in the SGZ. EdU incorporation was measured by counting the number of fluorescent cells in the SGZ at multiple timepoints including P5, P7, 1, 3 and 6 months of age (Figs. 4a, 4b). Quantification of EdU-positive cells revealed a consistent and significant reduction of proliferating cells in the SGZ of 3xTg mice as early as P7 (32.2 ± 12.8% reduction) and P5 (18.9 ± 8.4% reduction), as well as at later ages, 1, 3 and 6 months (Fig. 4b). To confirm these findings, we used another mitotic marker, the phosphor-histone H3 (pHH3), which revealed a similar trend detectable at P5 (Figs. 4c, 4d). Taken together, our data indicate that proliferation is compromised in the 3xTg SGZ starting from early postnatal ages and continuing into adulthood.

### Early transcriptional changes in the NSCs of 3xTg mice at 1 month of age

**Fig. 5:**
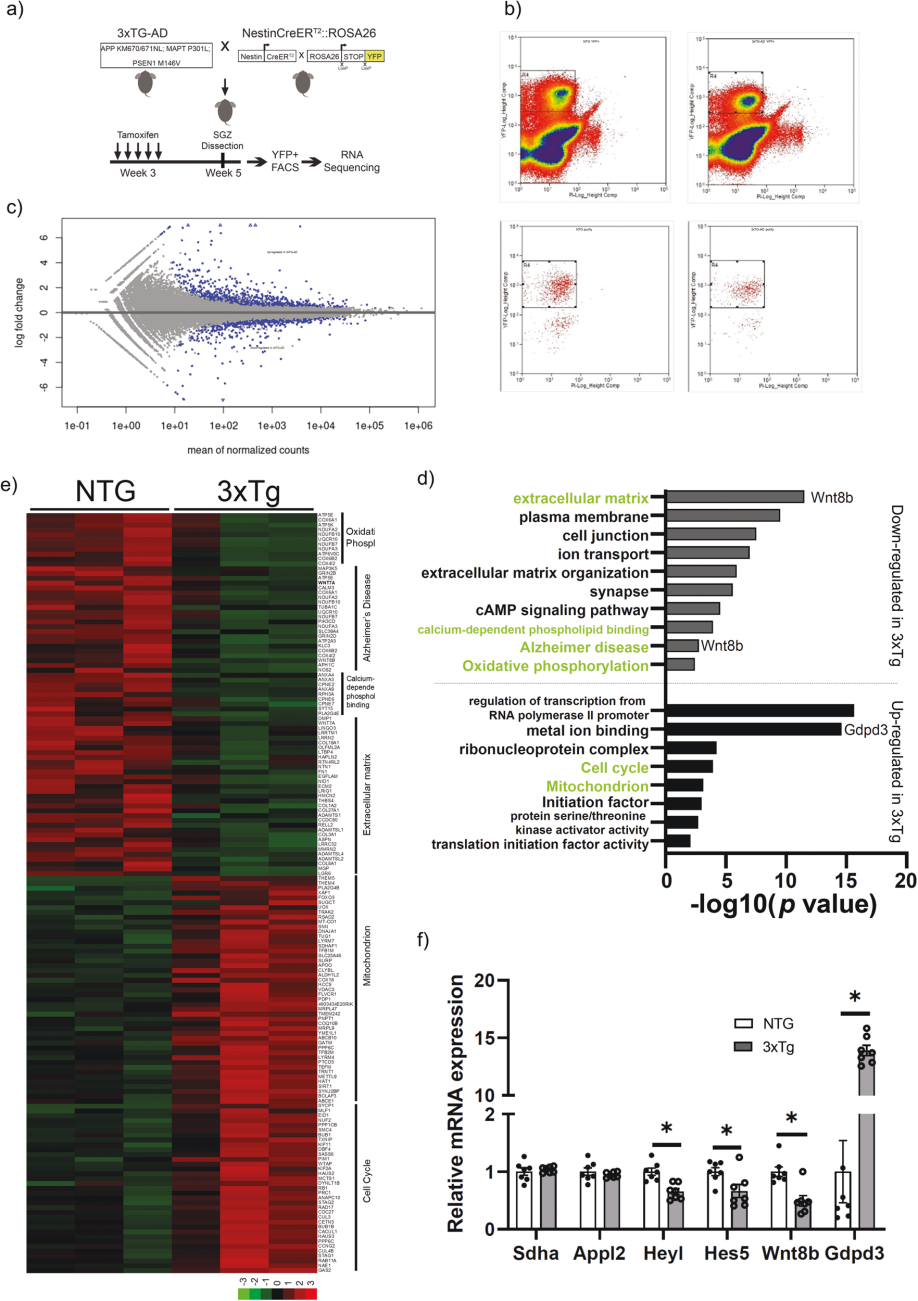
Early transcriptional changes in the SGZ of 3xTg at 1 month of age. **a** Methodology. **b** Cell sorting strategy of neural stem/progenitor cells using Nestin-YFP. **c** MA plot of RNA-seq. Significant genes are labelled in Blue with 1391 significant genes, 720 genes as down-regulated and 671 as up-regulated in the 3xTg cells. *n* = 3. **d** Gene ontology (GO) analysis of genes shown in **c**, up- or down- regulated respectively. **e** Heatmap showing the expression pattern of significantly altered genes grouped under different GO terms: Oxidative phosphorylation, mitochondrion, cell cycle, Alzheimer disease, calcium signaling, and extracellular matrix. Color density indicates the z-score computed from the normalized read counts. **f** RT-PCR validation of selected genes. Data represented as mean ± SEM. Statistical analysis was performed with an unpaired two-tailed Student’s *t*-test, *p* < 0.05*. *n* = 7.

Given the early defects in adult neurogenesis and in the neural stem/progenitor population, we asked if there were any significant changes in the transcriptome at early stages. Here, we used a tamoxifen (TAM)-inducible Nestin-CreER^T2^;R26LSL-EYFP^STOP^ reporter mouse line to label neural stem and progenitor cells in 3xTg and NTG control mice (17,18). YFP-positive cells from the SGZ of NTG and 3xTg brains were sorted through FACS at 1 month of age (pre-pathology), 10 days after TAM (Figs. 5a, 5b) [28]. Transcriptomic profiling revealed differential expression of 1391 genes in the 3xTg SGZ, with 720 down- and 671 up-regulated (Fig. 5c, d). Differentially expressed genes were classified into gene ontology category (GO term) (Fig. 5d). Our results revealed a number of transcriptional changes that were previously associated with neurodegeneration (Fig. 5e). Gdpd3, an enzyme involved in lipid metabolism and a marker for aging in the human brain, is significantly induced in 3xTg SGZ neural stem/progenitor cells [37] (Fig. 5f). Significant changes in pathways involved in stem cell homeostasis have also been identified in the 3xTg model. For example, Wnt8b, a canonical Wnt ligand, is down-regulated during SGZ neurogenesis in the 3xTg brain, where the Wnt signaling pathway was shown to be involved in neuronal plasticity, synaptic formation, and neuron maturation [38–40] (Fig. 5f). We also identified dysregulation of several members of the Notch signaling pathway including HeyL, which was shown to promote neuronal-specific differentiation, and Hes5, which is a key Notch effector important in neural stem cell homeostasis [41, 42]. Perturbations in these key pathways relevant to AD were confirmed by q-PCR in sorted YFP-positive cells from 1-month-old mice (Fig. 5f). Taken together, our results reveal very early perturbations in the SGZ neurogenic niche, manifested by cellular deficits and intrinsic molecular changes, resulting in dysregulated neural stem cell homeostasis and impaired neurogenesis at early postnatal ages in the 3xTg mouse model of AD.

## Discussion

The results of this study support the hypothesis of an early deficit in hippocampal neurogenesis in the 3xTg model of AD, as early as P0. The data highlight a reduction in the number of neural stem/progenitor cells as well as newborn neurons along with a decrease in the volume of hippocampal structures, first detected at P7. We report a consistent decrease in the number of Sox2/Hopx-expressing neural stem cells in early postnatal 3xTg mice that continues to 9 months of age. The number of newborn neurons (expressing Dcx) is also reduced in the 3xTg SGZ, declining sharply as animals approach adulthood (1 month old). The early postnatal deficits in hippocampal neurogenesis identified in the 3xTg mouse model, are accompanied by significant changes in the transcriptomic profiles of SGZ neural stem/progenitor cells, as early as one month of age. Genes involved in neurogenesis such as the Notch and Wnt pathways are dysregulated in the 3xTg mouse model.

A growing body of literature identified defects in human adult hippocampal neurogenesis that may contribute to the cognitive impairments related to AD [5, 10]. A recent study also shows that enhancing neurogenesis has the potential to rescue the cognitive decline observed in an AD mouse model [43]. These findings suggest an important link between AD and neurogenesis. Understanding the sequence of events leading to dementia is important for future diagnostic and therapeutic strategies [44].

### Volume reduction in hippocampal structures in early postnatal 3xTg mice

The results of this study support the hypothesis that defects in neurogenesis in the 3xTg model of AD occur early and precede the onset of AD pathology. When compared to NTG controls, 3xTg mice presented with a smaller volume of GCL, DG and hippocampus at both P7 and 3 months old (Fig. 1). This structural phenotype in the 3xTg model starts before the extracellular Aβ deposits and tangle formation which occur at the age of 6 months [45].

The dentate gyrus begins to form around E14–15.5 from neuroepithelial cells originating from the ventricular zone of the medial pallium. These cells proliferate and form intermediate progenitors and NSCs that delaminate and migrate toward the dentate fissure. The precursor cells will give rise to the GCL and the adult population of RGL [46, 47]. While dentate gyrus development begins prenatally, most of the granule neurons and the RGL are generated within the first postnatal week [36, 48]. Therefore, the neurogenesis defect we observed was at a critical period of SGZ development (P0, P5 and P7), and could explain the smaller GCL in 3xTg mice which then exacerbates throughout adulthood. We did not detect a change in the proliferating (EdU-positive) Hopx-positive neural precursor cells at E15.5 (Figure S1), indicating that defects in adult neurogenesis most likely arise postnatally.

### Reduction in the Hopx-positive population in the SGZ of 3xTg

A recent study showed through lineage tracing that in the adult DG, Hopx labels the RGL cells that have the potential to activate, self-renew, and differentiate into granule neurons [33]. Hopx-expressing population is reduced in the 3xTg model as early as P5, which is consistent with the proliferation and differentiation deficits detected at later stages.

The vast majority of neural stem/progenitor population in the dentate region were derived from Hopx-positive cells [33]. Hopx-positive population was not affected at E15.5 in the DNe and DMS (Fig. S1). However, this population was significantly decreased at P0. Combined with a reduction in Sox2/Hopx-positive cells in the 3xTg SGZ from postnatal period to adulthood, these data suggest that defects in the adult NSC may have originated neonatally [36, 46, 47].

### Early changes in the molecular signatures of SGZ neural stem/progenitor cells

We have demonstrated that 3xTg mice exhibit alterations in neurogenesis at early postnatal ages, however, the underlying mechanisms remain unknown. Previous studies have reported defects in lipid metabolism, mitochondrial function and energy metabolism in AD models [49–51]. However, many of these changes were observed in mice starting at 3 months of age. Thus, the mechanisms underlying the early impairments in neural stem/progenitor cell function remain unknown. To define the molecular changes behind the neurogenic deficits in the 3xTg animals, we performed bulk RNA-seq from Nestin-YFP labeled NSCs isolated directly from the brains of juvenile mice at 1 month of age. Interestingly, we found defects in multiple pathways including genes of the Notch pathway, Wnt pathway and those associated with mitochondrial function. The Notch pathway plays an important role in the maintenance of neural stem cell homeostasis [52, 53]. Dysregulation in Notch signaling was also proposed to contribute to cerebrovascular and AD pathophysiology associated with cognitive decline [54]. Both HeyL and Hes5, are basic helix-loop-helix (bHLH) transcription factors acting downstream of the Notch pathway. HeyL competitively activates pro-neuronal genes to promote neuronal differentiation [42]. Hes5, a transcriptional repressor, inhibits the expression of pro-neuronal genes and maintains the NSC pool [55, 56]. Down-regulation of HeyL and Hes5 may contribute to the neurogenesis deficits in the 3xTg SGZ. Similarly, two canonical Wnt ligands (Wnt7a and Wnt8b) are also downregulated (Fig. 5f). The Wnt signaling pathway has been shown to regulate neurogenesis in the developing and adult brain [38]. Interestingly, studies have reported a downregulation of Wnt signaling in AD patients [57]. Wnt7a is required for NSC maintenance, proliferation, differentiation and maturation [58]. Wnt8b was shown to act downstream of Sox21 during embryonic brain development [56].

Alzheimer’s disease is characterized by mitochondrial dysfunction as well as enhanced oxidative stress [59]. Studies from our group have shown that mitochondrial dysfunction, metabolic changes as well as the levels of reactive oxygen species can directly impact NSC fate decisions in the embryonic and adult brain [21]. In our RNA-seq analysis, we identified dysregulation of multiple genes involved in oxidative phosphorylation at as early as 1-month-old, suggesting that intrinsic defects in mitochondrial function may impact neurogenesis in the 3xTg SGZ.

Taken together, our data reveal significant changes in molecular pathways involved in the regulation of NSC proliferation and differentiation at very early ages in the hippocampus of 3xTg mice. Several cognitive tests have been developed to assess hippocampal function such as set-shifting tasks, pattern separation and memory tests with spatial navigation, which can serve as an early detection method [60–62]. The pathways identified in this study could serve as biomarkers of early AD detection which can collaborate with cognitive tests to help with early diagnosis, or even serve as potential therapeutic targets for early intervention.

## Supplementary information

Figure Legend S1

Figure S1

Table S1

Authorship file

Checklist

## Data Availability

The cell counts and volume measurements data used to support the findings of this study are available from the corresponding author upon request. RNA-seq data is deposited in Gene Expression Omnibus (GEO) database (GSE218323).
