# Supplementary material for: Early postnatal defects in neurogenesis in the 3xTg mouse model of Alzheimer’s disease
Source: Cell Death Dis. 2023 Feb 18;14(2):138. doi: 10.1038/s41419-023-05650-1 (PMC9938901; doi:10.1038/s41419-023-05650-1)
Supplement: Supplementary file 1 — Figure Legend S1 [file 41419_2023_5650_MOESM1_ESM.docx]

**Supplementary Figure S1. Characterizing the Hopx/EdU-positive cell population in the DNe and DMS at E15.5.**

**a)** Representative fluorescent images of Hopx and EdU staining in the DNe and DMS after 24 hours of incorporation. Scale bar 100*µm*. Quantification of total Hopx-positive cells **b)**, Hopx/EdU-positive cells **c)**, and percentage of EdU-positive cells in Hopx-expressing population **d)** in the DNe and DMS of NTG and 3xTg mice at E15.5.
