# Supplementary material for: Early postnatal defects in neurogenesis in the 3xTg mouse model of Alzheimer’s disease
Source: Cell Death Dis. 2023 Feb 18;14(2):138. doi: 10.1038/s41419-023-05650-1 (PMC9938901; doi:10.1038/s41419-023-05650-1)
Supplement: Supplementary file 4 — Authorship file [file 41419_2023_5650_MOESM4_ESM.docx]

Smitha Paul <spaul@uottawa.ca>

Wed 01/02/2023 15:04

To:

- Yubing Liu <bingliusong@gmail.com>;
- Maria Bilen <mbile027@uottawa.ca>;
- Marie-Michelle McNicoll <mmcni102@uottawa.ca>;
- Marie-Michelle.mcnicoll@mail.mcgill.ca <Marie-Michelle.mcnicoll@mail.mcgill.ca>;
- Richard Harris <rharris3@gmail.com>;
- Bensun Cambell Fong <bfong099@uottawa.ca>;
- Mohamed Ariff Iqbal <miqba095@uottawa.ca>;
- Janice Mayne <jmayn2@uottawa.ca>;
- Krystal Walker <kwalker2@uottawa.ca>;
- Jing Wang <JIWang@ohri.ca>;
- Daniel Figeys <dfigeys@uottawa.ca>;
- Ruth Slack <rslack@uottawa.ca>

Hello Everybody,

                   We have submitted the revised manuscript, and the quality check is in process.  In order for the process to finish we have to submit one more document which is the change in authorship. Because the author list differs from our original submission, can you please reply to this email confirming that you agree to the new authorship list? Please find below the authors list as submitted in the revised manuscript.

**Early Postnatal Defects in Neurogenesis in the 3xTg Mouse Model of Alzheimer’s Disease**

**Yubing Liu^1^*, Maria Bilen^1^*, Marie-Michelle McNicoll^1^, Richard A. Harris^1^, Bensun C. Fong^1^, Mohamed Ariff Iqbal^1^, Smitha Paul^1^, Janice Mayne^2^, Krystal Walker^2^, Jing Wang^1^, ^3^, Daniel Figeys^2^, and Ruth S. Slack^1^**

1 Department of Cellular and Molecular Medicine, University of Ottawa Brain and Mind Research institute, Ottawa, Canada, K1H 8M5.

2 Ottawa Institute of Systems Biology and Department of Biochemistry, Microbiology and Immunology, Faculty of Medicine, University of Ottawa, Ottawa, Canada, K1H 8M5

3 Regenerative Medicine Program, Ottawa Hospital Research Institute, Ottawa, Canada, K1H 8L6.

*Both authors contributed equally

**Re: CDDIS-22-0428RR**

**Yubing Liu <bingliusong@gmail.com>**

Wed 01/02/2023 15:26

To:

- Smitha Paul <spaul@uottawa.ca>

Yes, I agree.

**Maria Bilen <mbile027@uottawa.ca>**

Wed 01/02/2023 15:38

I agree

Maria

**Marie-Michelle McNicoll <marie-michelle.mcnicoll@mail.mcgill.ca>**

Wed 01/02/2023 15:40

I agree.

MMM

**Richard Harris <rharris3@gmail.com>**

Wed 01/02/2023 15:19

To:

- Smitha Paul <spaul@uottawa.ca>

Looks good to me, thanks Smitha

**Bensun Cambell Fong <bfong099@uottawa.ca>**

Wed 01/02/2023 15:05

To:

- Smitha Paul <spaul@uottawa.ca>

Confirming agreement! Way to go!

Sincerely,

Bensun

—
**Dr. Bensun C. Fong, PhD**(he, him)

**Mohamed Ariff Iqbal <miqba095@uottawa.ca>**

Wed 01/02/2023 15:27

    I agree with the authorship order.

Sincerely,

Ariff

**Smitha Paul(**[**spaul@uottawa.ca)**](mailto:spaul@uottawa.ca))

I agree

**Janice Mayne <jmayn2@uottawa.ca>**

Wed 01/02/2023 15:35

Yes, I agree.

Regards,

Janice

**Krystal Walker <kwalker2@uottawa.ca>**

Thu 02/02/2023 12:07

I agree to the new author list.

Krystal Walker RLAT RVT

**Jing Wang <JIWang@ohri.ca>**

Wed 01/02/2023 16:43

I agree, too.

Jing

**Daniel Figeys <dfigeys@uottawa.ca>**

Wed 01/02/2023 15:27

I also agree.

Daniel

**Ruth Slack <rslack@uottawa.ca>**

Wed 01/02/2023 15:46

I agree.

Thanks, Ruth
